# A Stereo Dual-Channel Dynamic Programming Algorithm for UAV Image Stitching

**DOI:** 10.3390/s17092060

**Published:** 2017-09-08

**Authors:** Ming Li, Ruizhi Chen, Weilong Zhang, Deren Li, Xuan Liao, Lei Wang, Yuanjin Pan, Peng Zhang

**Affiliations:** 1 State Key Laboratory of Information Engineering in Surveying Mapping and Remote Sensing, Wuhan University, Wuhan 430079, China; lisouming@whu.edu.cn (M.L.); drli@whu.edu.cn (D.L.); sdliaoxuan@163.com (X.L.); lei.wang@whu.edu.cn (L.W.); pan_yuanjin@163.com (Y.P.); fenix@whu.edu.cn (P.Z.); 2 Collaborative Innovation Center of Geospatial Technology, Wuhan University, Wuhan 430079, China; 3 School of Resource and Environmental Science, Wuhan University, Wuhan 430079, China

**Keywords:** UAV image, dual-channel, dynamic programming, seam line, energy function

## Abstract

Dislocation is one of the major challenges in unmanned aerial vehicle (UAV) image stitching. In this paper, we propose a new algorithm for seamlessly stitching UAV images based on a dynamic programming approach. Our solution consists of two steps: Firstly, an image matching algorithm is used to correct the images so that they are in the same coordinate system. Secondly, a new dynamic programming algorithm is developed based on the concept of a stereo dual-channel energy accumulation. A new energy aggregation and traversal strategy is adopted in our solution, which can find a more optimal seam line for image stitching. Our algorithm overcomes the theoretical limitation of the classical Duplaquet algorithm. Experiments show that the algorithm can effectively solve the dislocation problem in UAV image stitching, especially for the cases in dense urban areas. Our solution is also direction-independent, which has better adaptability and robustness for stitching images.

## 1. Introduction

Unmanned aerial vehicle (UAV) remote sensing systems have been widely used in environmental monitoring, ecological farming, disaster emergency management, navigation map production, and 3D urban reconstruction because of its advantages of low cost, fast data collection, and easy operation [1,2,3,4,5]. Due to its low flight altitude and the camera perspective constraints, the coverage area of a single UAV image is small. In order to expand the image coverage area to capture more information from the target area, multiple UAV images are collected, leading to the need of stitching multiple images to form a mosaic image. Because high-altitude wind has a significant impact on the UAV platform due to its light-weight [6], problems such as irregular image overlapping and uneven image exposure are introduced into the adjacent images. Furthermore, images captured from an unstable UAV platform will lead to a vulnerable stitched image with ghosting, blur, dislocation, and color inconsistence.

Various methods for seamless stitching of UAV remote sensing images have been investigated [7,8,9,10,11,12,13,14,15,16,17]. These methods can generally be classified into two types: weighted fusion algorithms and seam line-based algorithms [7,8,9,10,11,12,13,14]. The weighted fusion algorithms focus on overlapping area pixels on the adjacent images. They adopted algorithms to eliminate the seams, which can be simply and easily implemented and can effectively adjust the exposure difference [7,8,9,10]. These weighted fusion algorithms process the pixels directly into the spatial domain of the images. However, when there are matching errors or abundant image texture structures in the overlapping area, the stitched images will produce ghosting, dislocation, and fuzzy phenomena and weak texture details [7,8,9,10]. Seam line-based algorithms are intended to reduce grayscale and geometric differences, and consist of energy function-based algorithms (including the dynamic programming algorithm, Graph cut-based algorithm) and geometric information-based algorithms [11,12,13,14]. The latter have the advantages of being fast and having simple calculation, and do not have the requirement of constructing an energy function. In contrast, the former focusing on the energy difference between the images and its effect is superior. Additionally, energy function-based algorithms [11,12,13] adopt Dijkstra’s shortest path algorithm to search for the optimal seam line, which addresses the ghosting and dislocation problems because of the movements of the objects and registration errors, but it suffers from low search efficiency and complex weight determination. The seam line-based algorithms—more specifically, the ant colony algorithm—can evade the area where the color contrast is larger on the image, while it will easily lead the search processing to the local optimum due to its sensitivity to ants’ number [14]. Moreover, there are some algorithms based on the snake model [15], and some based on morphological model [16,17]. Although these algorithms can almost guarantee the consistence of the geometric structure and evade the phenomenon of ghosting in overlapping areas under certain conditions, they are unable to guarantee that ghosting and seams will be overcome at the same time—especially when there is a significant brightness difference in the adjacent images. Similarly, these algorithms are unable to achieve satisfactory results when there are rich texture structures in image pairs, registration errors, and radiation brightness differences. Furthermore, most of the current seam line generation methods do not adopt the dynamic programming algorithm. This is because the dynamic programming algorithm is relatively mature in theory, and not easy to develop further. Furthermore, it relies strongly on image direction that leads to a low robustness with modified energy functions. 

This paper proposes a new algorithm to realize the seamless stitching of UAV images through a comprehensive theoretical analysis of the dynamic programming algorithm. The algorithm especially solves the dislocation problem caused by the seam line in the building-intensive areas. The basic idea is to determine the geometric errors introduced by perspective errors, camera distortions, and radiation errors by analyzing the mapping relationships between the left and right images. The determination of these errors can aid the seam line searching process. Thus, it is an optimization problem. There is no unique (that is a non-ground-truth problem) solution for this image seam line searching problem. Fortunately, we only require a close optimum solution that meets the requirement of stitching the images. 

## 2. Methodology 

### 2.1. Duplaquet Algorithm

In 1958, Bellman proposed an optimization solution that transforms the multi-stage process into a series of single-stage processes, and thus he invented the dynamic programming algorithm [18]. Based on this, Duplaquet proposed a classic algorithm for searching image seam lines. The algorithm ensures that the lengths of all alternative seam lines are equal, and the seam line with the smallest accumulated energy value is the optimal seam line. Equation (1) shows the energy criterion defined in the algorithm [19]:

*C*(*x*, *y*) = *C_dif_* (*x*, *y*) − λ*C_edge_* (*x*, *y*), (1)

where *C_dif_* (*x*, *y*) is the mean value of the gray level difference of the pixel in the overlapping areas between two adjacent images, *C_edge_* (*x*, *y*) is the minimum gradient value of the pixel in the overlapping areas of the image pair, and λ is a weighing factor, which can be used to adjust the proportion of gray difference and structure difference in energy function.

### 2.2. Problems Analysis

**Figure 1:**
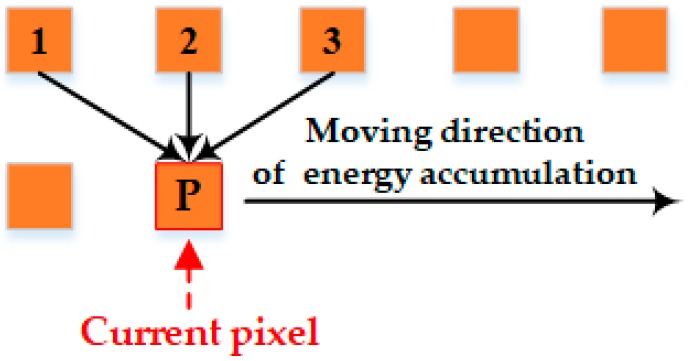
The schematic diagram of Duplaquet’s energy criterion.

**Figure 2:**
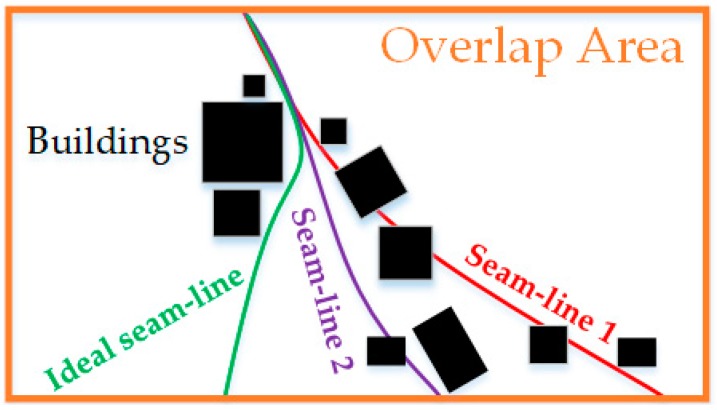
The sketch map of search results for seam lines.

The energy criterion in the Duplaquet algorithm only considers the horizontal and vertical gradients, and compares the pixels in three adjacent directions near the current pixel, as shown in Figure 1. When the overlapping area has dense high-rise buildings and tall trees, the seam lines output from the Duplaquet algorithm are likely across the edge of the buildings due to the inconsistent deformation from image point to the roof point. Therefore, it is easy to find the dislocations in the stitched image leading to a large matching error. Furthermore, it is not guaranteed to obtain the best seam line by using the classical Sobel operator to calculate the approximate gradient of the pixel based on the horizontal and vertical templates (see Equation (2)) without considering diagonal directions in the calculation process [20]. As shown in Figure 2, the red seam-line not only crosses the edge of the houses, but also deviates from the ideal seam-line.

(2) $$D_{x}=\left[\begin{array}{ccc} -1 & 0 & 1\\ -2 & 0 & 2\\ -1 & 0 & 1 \end{array}\right]\;D_{y}=\left[\begin{array}{ccc} -1 & -2 & -1\\ 0 & 0 & 0\\ 1 & 2 & 1 \end{array}\right]$$

Specifically, the algorithm has the following main problems: (1) The gradient guidance direction of the energy function does not support omnidirectional searching. (2) The energy function is direction-dependent; the energy aggregation takes only three directions into consideration, and the direction of energy traversal is also limited from left to right, as well as from top to bottom. (3) The energy function is prone to local optimal solution due to the impact of the two factors mentioned above. This will directly lead to the situation where the optimal seam line can be easily affected by buildings.

**Figure 3:**
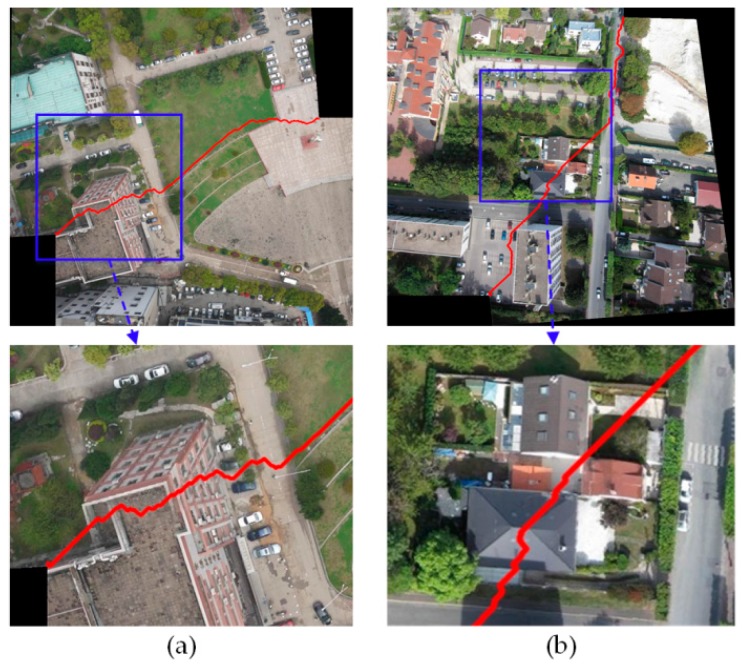
The stitching results using the Duplaquet algorithm for two datasets. (**a**) The experimental result with UAV images shot in Wuhan; (**b**) The experimental result with UAV images shot in Paris.

There are two experimental results based on the Duplaquet algorithm. As shown in Figure 3, the seam lines across the houses are prone to ghosting and dislocation in stitched images. In addition, some researchers believe that the energy function is poorly fitted, making it difficult to find the optimal seam line. For this reason, these researchers attempt to modify the energy function in dynamic programming; however, they overlook the optimality of the corresponding model. This also happens in the algorithms included in the OpenCV library.

### 2.3. Our Algorithm

We developed a new algorithm for searching seam line by taking gradient guidance direction, energy accumulation directions (energy aggregation direction, energy traversal direction), and energy local optima into account.

#### 2.3.1. Gradient Calculation

The Duplaquet algorithm only considers the horizontal and vertical gradient in the energy criterion; it fails to obtain the optimal seam line in some cases. In order to solve this problem, we develop a new algorithm on the basis of the classical Sobel operator, by considering eight directional neighborhood information of current pixel and the similarity of its surrounding structure [21]. The new approach of gradient calculation is as follows:

(3) D0°=[121000−1−2−1]Dπ4=[21010−10−1−2]Dπ2=[10−120−210−1]D3π4=[0−1−210−1210]Dπ=[−1−2−1000121]D5π4=[−2−10−101012]D3π2=[−101−202−101]D7π4=[012−101−2−10]

#### 2.3.2. Directionality of Energy Accumulation

**Figure 4:**
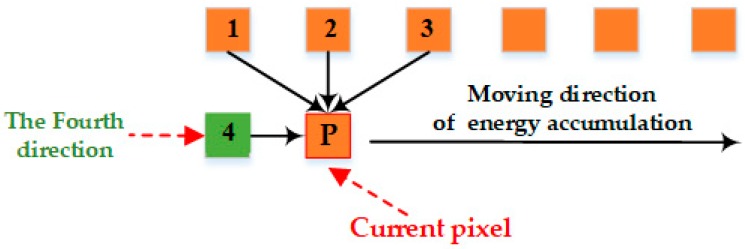
Schematic diagram of energy accumulation by improving energy guidelines.

In order to solve the direction-dependent problem in energy accumulation, we introduce a new direction in our algorithm, as shown in Figure 4. The algorithm resolves the problem of seam line deviation from the true seam line with an improved strategy of energy aggregation. As shown in Figure 2, the improved purple seam-line 2 is closer to the ideal seam line than the red seam line 1, despite its obvious insufficiency.

**Figure 5:**
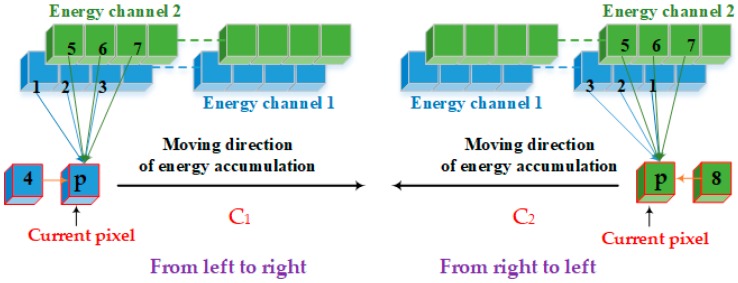
Schematic diagram of our search strategy.

The optimal solution of the energy function is affected by not only the directions of energy aggregation, but also the directions of energy traversal. Therefore, we redefined the energy criterion and added the aggregate directions to our new dynamic programming algorithm with a stereo dual-channel energy accumulation strategy. It improves the searching scheme of optimal seam line based on energy accumulation. As shown in Figure 5, there is a schematic diagram of our optimal seam line search strategy, which optimizes the seam line search criteria by detecting the eight pixels (contain the horizontal direction) surrounding the current pixel.

In Figure 5, **P** is the current pixel. We redefine the nine related directions surrounding **P** as follows: 0 (initial invalid direction), 1 (top-left of the current pixel for energy aggregation channel 1), 2 (top of the current pixel for energy aggregation channel 1), 3 (top-right of the current pixel for energy aggregation channel 1), 4 (left of the current pixel for energy aggregation channel 1), 5 (top-left of the current pixel for energy aggregation channel 2), 6 (top of the current pixel for energy aggregation channel 2), 7 (top-right of the current pixel for energy aggregation channel 2), 8 (right of the current pixel for energy aggregation channel 2). Seam line searching is an aggregation process of minimum energy. Each seam line consists of neighborhood pixels with smallest energy value. In our approach, the longest seam line is the optimal seam line. Furthermore, it requires the shape of the overlapping area and close distance to be adapted to the center line of adjacent images.

#### 2.3.3. The Energy Function

Based on the analysis of the theoretical model, we constructed a mathematical abstract expression of the theoretical model. Assuming that image 1 and image 2 are an original image pair to be stitched, the energy function is defined as follows:

(4) $$\begin{array}{l} E=\sum_{(x,y)\in O}B(x,y)\sigma (|f_{1}(x,y)-f_{2}(x,y)|)+\\ \sum_{(x,y)\in O}\sigma \left(\max\left(\left|\underset{0\leq k\leq 7}{d_{k}}(f_{1}(x,y))-\underset{0\leq k\leq 7}{d_{k}}(f_{2}(x,y))\right|\right)\right)+\sum_{(x,y)\notin O}N(x,y) \end{array}$$

In Equation (4), *B*(*x*, *y*) determines whether the current pixel (*x*, *y*) is in the boundary of overlapping area of the adjacent images; when *B*(*x*, *y*) = 1, it means that it is not in the boundary region, and when *B*(*x*, *y*) = 10, it is in the boundary region. σ(*) is the Gaussian smoothing term, which uses the information in the local window to enhance the local influence of the current pixel; *f*_1_(∙), *f*_2_(∙) are pending images to be stitched; ***O*** is the overlapping area; *d*(*) represents the gradient function of one of the eight directions; *N*(*x*, *y*) is the energy value of the invalid area, which is the constant term, and the value is 100 times larger than the maximum value of ***O***.

#### 2.3.4. Computation Procedure

**Figure 6:**
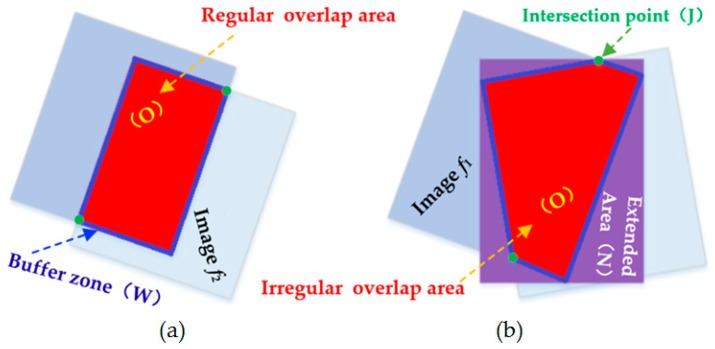
Processing principle of different images overlap area: (**a**) Regular overlap area; (**b**) Irregular overlap area.

The image size of the overlapping area is set as *m* × *n*, and if the overlapping area is irregular, it can be extended to a regular area by using the minimum exterior rectangle of the overlapping area as shown in Figure 6.

The image energy matrix ***E*** can be obtained by Equation (4). The algorithm procedure is as follows:

(1) Extract the corresponding points from the adjacent images in order to correct the left and right pending matching images to the virtual unified reference image, so that the images are in the same coordinate system.
(2) Define the overlapping area of the adjacent images ***O***, the boundary buffer area ***W*** (set its width value is 20 pixels), and ***W*** is an empirical value, the invalid area ***N*** (extend area), and the boundary intersection ***J***.
(3) Calculate the matrices ***O***, ***W***, ***N*** according to Equation (4), where ***W*** ∈ [1, 10], the closer to the boundary, the larger the value is, and set ***N*** = 100 × max(***O***); ***J*** = −1000 × max(***O***).
(4) Fill the energy matrix ***E*** according to the results of (3).
(5) Reestablish two energy aggregation channels: the ***C****1* and ***C****2* matrices, which have the same size as ***E***; each pair of corresponding elements in these two matrices hold two scalar numbers representing the current aggregation value and the current path direction of the seam line.
(6) For the first row of the energy aggregation channel matrix assigned with the first row of ***E*** as the initial value, set its corresponding direction as 0.
(7) The energy aggregation channel matrix starts to make a difference from the second row, which is divided into two aggregation processes from left to right and from right to left. For the energy aggregation channel ***C****1*, its aggregation process is from the left to the right; the current pixel only considers the directions of 1, 2, 3, 4, 5, 6, and 7. For the energy aggregation channel ***C****2*, its aggregation process is from the right to the left, and the current pixel only considers the directions of 1, 2, 3, 5, 6, 7, and 8.
(8) When the aggregation is completed, the minimum energy value is found from the last row in ***C****1* and ***C****2* respectively; an optimal stitching path is then found based on the direction information stored in the matrixes.

In order to ensure that the seam line starts and ends at the intersection points, we select two special intersection points that have the smallest energy value, so that the seam line can be guided and adsorbed.

## 3. Experiments and Analysis

### 3.1. Experimental Environment and Data

**Figure 7:**
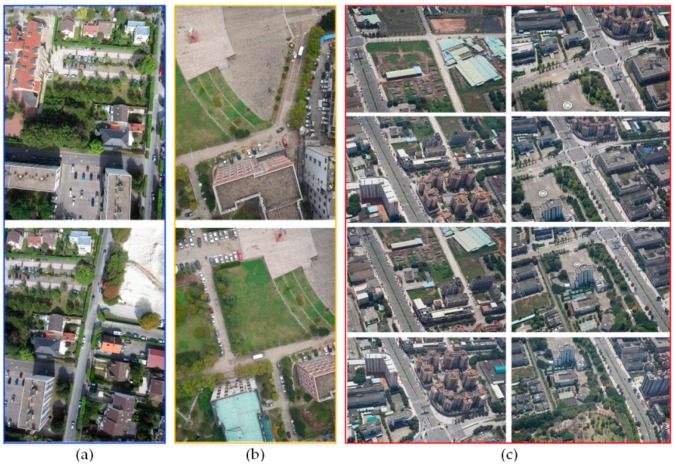
Three groups of experimental unmanned aerial vehicle (UAV) images. (**a**) The first image pair; (**b**) The second image pair; (**c**) The sequence images of a single-strip.

**Figure 8:**
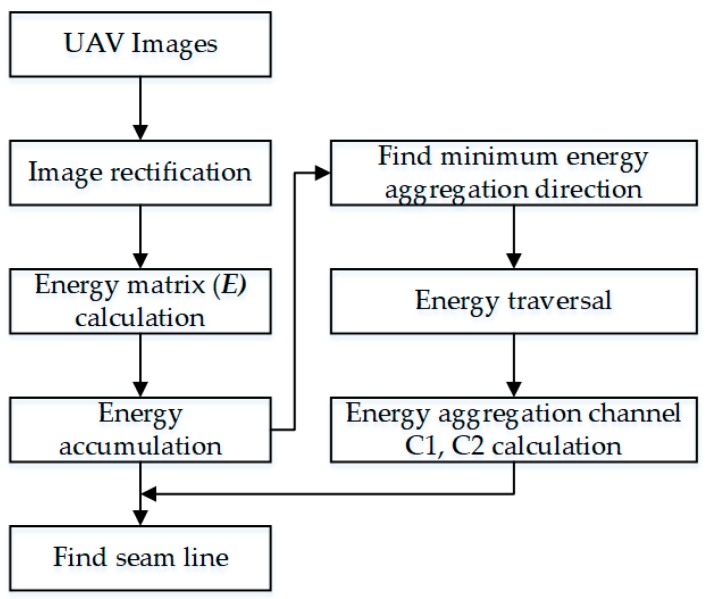
A flow chart of our algorithm.

To verify the effectiveness of our algorithm, we not only utilized the UAV images from different regions with different flight altitudes, but also compared the processing results with the Duplaquet algorithm and the algorithms from open source in OpenCV. In this paper, we used Visual C++ based on OpenCV open source library to program the proposed improvement algorithm. The experimental images are divided into three groups; among them, the data in Figure 7a were acquired by Canon IXUS 220HS (Canon, Oita, Japan) in Paris, its focal length is 4 mm, and the height of the UAV was approximately 250 m. The data in Figure 7b were acquired by DMC-GF3 (Panasonic, Xiamen, China) at Wuhan University Square, its focal length is 14 mm, and the height of the UAV was approximately 200. The data in Figure 7c are a sequence of images of a single-strip; they were acquired by ILCE-600 (Sony, Chonburi, Thailand) in Shaxi town, China, its focal length is 35 mm, and the height of the UAV was approximately 410–440 m. The experimental computer environment is Windows 7 operating system, CPU: Intel (*R*) Core (*TM*) i7-4790, RAM: 32 GB. Figure 8 is a flow chart of our algorithm, listing the key steps of the algorithm.

### 3.2. Results Analysis

#### 3.2.1. Comparison of Three Algorithms under the Condition of Image Rotation

**Figure 9:**
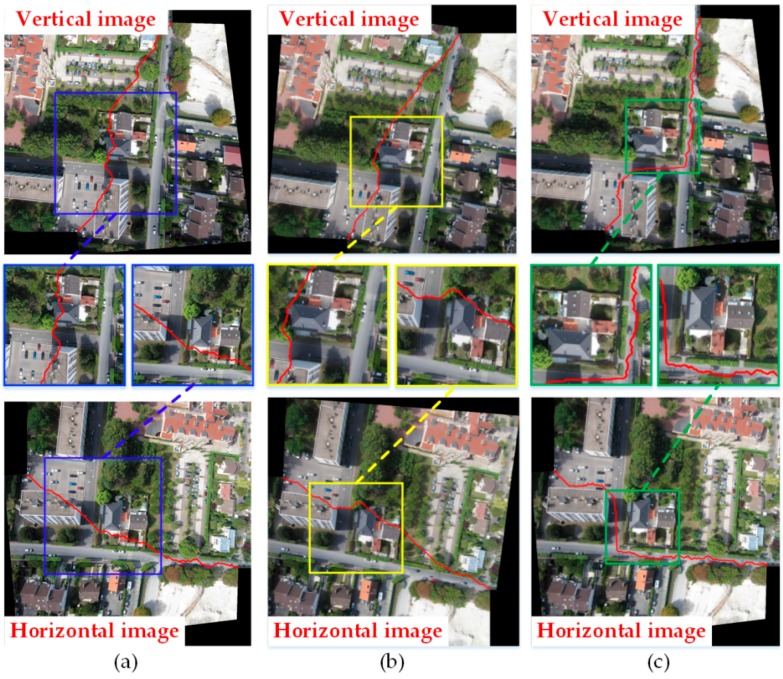
The seam lines of different searching algorithms under different situations. (**a**) Duplaquet algorithm; (**b**) OpenCV algorithm; (**c**) Our algorithm.

Firstly, the vertical images in Figure 7a were rotated to be horizontal. Then, we used the Duplaquet algorithm, the OpenCV algorithm, and the algorithm proposed in this paper to search the seam lines. Figure 9 shows the results. The partially enlarged pictures illustrated that the Duplaquet algorithm changed the search results of the optimal seam lines before and after rotation, and the seam lines passed through the edge of buildings in two cases. The optimal seam lines searched by the OpenCV algorithm had slight changes before and after rotation. The seam lines searched by the Duplaquet algorithm and the OpenCV algorithm crossed the edge of the houses in different locations. Furthermore, there was also a problem of house information loss in stitched images. The seam lines searched by our algorithm had basically no change before and after rotation; they still went along the road, and were very good at avoiding the ground buildings. This shows that the traditional methods are sensitive to the direction of images, and the direction of seam lines change with the direction of images. That is to say, the minimum value of the energy function is related to the direction of energy aggregation and traversal, and they may also lead to house information loss. Therefore, due to the specific improvements to the above issues, our algorithm has advantages in adaptability and robustness for different UAV images.

#### 3.2.2. Comparison of Stitching Results with Two Groups of Image Pairs

**Figure 10:**
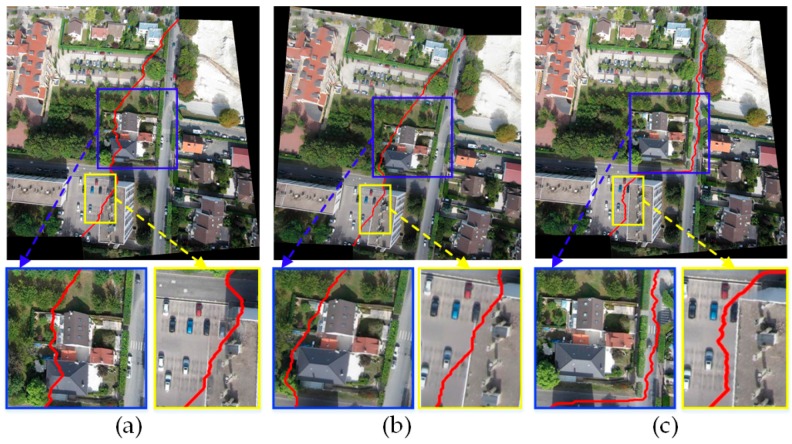
The seam lines of different searching algorithms for Figure 7a: (**a**) Duplaquet algorithm; (**b**) OpenCV algorithm; (**c**) Our algorithm.

**Figure 11:**
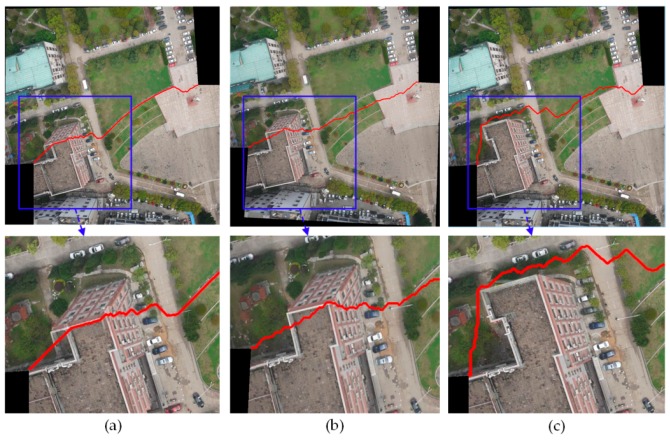
The seam lines of different searching algorithms for Figure 7b: (**a**) Duplaquet algorithm; (**b**) OpenCV algorithm; (**c**) Our algorithm.

In order to further verify the superiority of our algorithm, the Duplaquet algorithm, the OpenCV algorithm, and our algorithm were used to search the optimal seam lines of image pairs in Figure 7a,b with irregular overlapping areas. Figure 10 and Figure 11 are the respective results. It can be seen from Figure 10 and Figure 11 that the seam lines are obviously different with the three test algorithms. From the local zoom view of Figure 10 and Figure 11, we can find that the Duplaquet algorithm not only places the seam lines across the edge of houses, but also presents a ghosting phenomenon. The OpenCV algorithm often focuses on making the seam line follow along the edge of buildings as much as possible to conceal the seam lines, but the seam lines are still prone to cross the edge of ground buildings. Besides, the house corner information is lost in Figure 10 and Figure 11. In this paper, the optimal seam lines searched by our algorithm are basically following along the road direction, which avoids the ground buildings; this will greatly reduce the probability of dislocation and the seams reason of image geometric errors. The other two algorithms are prone to stitching dislocation, ghosting, and image information loss around the edge. 

#### 3.2.3. Stitching with an Image Sequence

**Figure 12:**
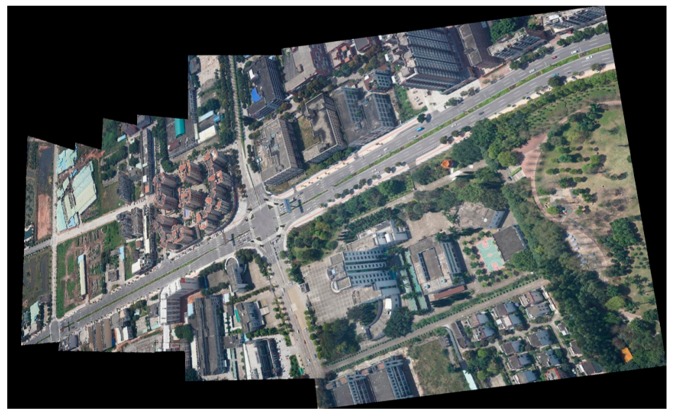
The stitching result of a single-strip image sequence.

In this experiment, we selected eight UAV images in a single-strip image sequence. Figure 12 showed the results of the single-strip image sequence by the algorithm proposed in this paper. We can see that the algorithm achieved a good result for the stitching of multiple UAV images. There is basically no ghosting, seams, or dislocation in the stitched panorama image. This further illustrates that a strategy based on finding an optimal seam line can avoid crossing the ground buildings, so it is a good solution to UAV image stitching.

#### 3.2.4. Comparing the Efficiency of Energy Accumulation Processing

In the previous experimental results, our algorithm found the most satisfying seam lines. Since this algorithm is based on the classical Duplaquet algorithm, this section will compare the energy accumulation time efficiency of these two algorithms. Firstly, we assumed that our algorithm and the Duplaquet algorithm could find the same optimal seam line. Their time efficiency difference can be quantitatively analyzed from the algorithms’ complexity. In this paper, the direction of energy aggregation from three aggregation directions increased to eight is mainly an improvement. Assuming that the time complexity of the Duplaquet algorithm is ***O***(***m***^3^) and the time complexity of our algorithm is ***O***(***m***^8^), where ***m*** is the total number of pixels within the minimum exterior rectangle of overlapping image area, ***m*** can be expressed as the product of ***w*** and ***h***, where ***w*** is the width of the minimum exterior rectangle and ***h*** is the height of the minimum exterior rectangle. Both ***w*** and ***h*** are measured by unit pixel. However, because the local energy minima exists in the energy function of the Duplaquet algorithm, it results in a lot of time consumption. So, the above assumption is invalid.

We selected five experimental image pairs to verify the above conclusions. Image pair 1 came from Paris, image pair 2 came from Wuhan University, image pairs 3–5 came from Shaxi town. In order to speed up the calculation, it is generally necessary to zoom the image at a certain scale. So, the size of the overlapping area is not the size of the original image overlap area. The experimental results can be seen in Table 1. The efficiency of our algorithm is more than 40 times that of the Duplaquet algorithm. The convergence speed of the energy function is faster. It is necessary to further point out that the theory and the results of the proposed algorithm are obviously different than the classical Duplaquet algorithm. The theoretical improvement and experimental comparisons has proven that the Duplaquet algorithm has a serious local optimal problem and a direction-sensitive problem; they only have the same observation conditions and common goal. The results of the Duplaquet algorithm are a pseudo-solution, and the results of the two algorithms are the same only in the intersection of their feasible solutions, but this is a small probability event. In addition, this paper proposed a global and non-direction optimization algorithm, which not only has the optimal seam line, but also has better time efficiency.

## 4. Conclusions

This paper analyzed and evaluated the current mainstream stitching algorithms for the problems of ghosting, dislocation, and seams in UAV image stitching. Then, it selected the essential problems of dynamic programming algorithms for seam line searching, and carried out a detailed theoretical study and a large number of UAV image stitching experimental verifications. At last, this paper proposed a stereo dual-channel dynamic programming algorithm to search the optimal seam lines through several improved key problems of the classical Duplaquet algorithm. Meanwhile, the superiority and efficiency of the algorithm proposed in the paper are verified by the credible experiments of two image pairs and an image sequence with irregular overlapping area. The stitched results are better than the Duplaquet algorithm and the OpenCV algorithm. The proposed algorithm is proved to be direction-independent, more efficient, and more robust. However, this paper did not consider the role of image structure information in stitching too much, and that is our next research direction to consider the optical flow in dynamic programming algorithm for UAV image stitching.

## Figures and Tables

**Table 1:** Compared time efficiency of energy accumulation processing.

Image Pair	1	2	3	4	5
*m* = *w* × *h*	957 × 523	909 × 421	919 × 317	923 × 384	919 × 335
Location	(Paris)	(Wuhan University)	(Shaxi town)	(Shaxi town)	(Shaxi town)
Duplaquet algorithm	4898 ms	3572 ms	2735 ms	3368 ms	2854 ms
Our algorithm	91 ms	69 ms	54 ms	68 ms	54 ms
Multiple	53	51	50	49	52

## References

[1] Chen S., Laefer D., Mangina E. (2016). State of technology review of civilian UAVs. Recent Pat. Eng..

[2] Byrne J., Keeffe E., Lenon D., Laefer D. (2017). 3D reconstructions using unstabilized video footage from an unmanned aerial vehicle. J. Imaging.

[3] Li D., Li M. (2014). Research advance and application prospect of unmanned aerial vehicle remote sensing system. Geomat. Inf. Sci. Wuhan Univ..

[4] Chen R., Chu T., Landivar J., Yang C., Maeda M. (2017). Monitoring cotton (*Gossypium hirsutum* L.) germination using ultrahigh-resolution UAS images. Precis. Agric..

[5] Zhang W., Li M., Guo B., Li D., Guo G. (2017). Rapid texture optimization of three-dimensional urban model based on oblique images. Sensors.

[6] Li M., Li D., Fan D. (2012). A study on automatic UAV image mosaic method for paroxysmal disaster. Int. Arch. Photogramm. Remote Sens. Spat. Inf. Sci..

[7] Wang W., Michale K. (2013). A variational approach for image stitching. SIAM J. Imaging Sci..

[8] Tao M., Johnson M., Paris S. (2013). Error tolerant image compositing. Int. J. Comput. Vis..

[9] Levin A., Zomet A., Peleg S. Seamless image stitching in the gradient domain. Proceedings of the European Conference on Computer Vision.

[10] Zomet A., Levin A., Peleg S., Weiss Y. (2006). Seamless image stitching by minimizing false edges. IEEE Trans. Image Process..

[11] Dijkstra E. (1995). A note on two problems in connexion with graphs. Numer. Math..

[12] Davis J. Mosaics of scenes with moving objects. Proceedings of the IEEE Computer Society Conference on Computer Vision & Pattern Recognition.

[13] Chon J., Kim H., Lin C. (2010). Seam-line determination for image mosaicking: A mismatch and the global cost. ISPRS J. Photogramm. Remote Sens..

[14] Zhang J., Sun M., Zhang Z. (2009). Automated seamline detection for orthophoto mosaicking based on ant colony algorithm. Geomat. Inf. Sci. Wuhan Univ..

[15] Gracias N., Mahoor M., Negahdaripour S., Gleason A. (2009). Fast image blending using watersheds and graph cuts. Image Vis. Comput..

[16] Bielski C., Grazzini J., Soille P. Automated morphological image composition for mosaicking large image data sets. Proceedings of the IEEE International Geoscience and Remote Sensing Symposium.

[17] Soille P. (2006). Morphological image compositin. IEEE Trans. Pattern Anal. Mach. Intell..

[18] Bellman R. (1958). On a routing problem. Q. Appl. Math..

[19] Duplaquet L. (1998). Building large images mosaics with invisible seam-lines. Proc. SPIE.

[20] Gonzalez R., Woods R. (2011). Digital Image Processing.

[21] Cheng X. (2011). Research on Fast Produce of Orthophoto with UAV Sequence Images.

